# Demographic Pattern and Mortality Risk Factors for Prescription Opioid Overdose Hospitalizations: Results From Nationwide Inpatient Sample Analysis

**DOI:** 10.7759/cureus.15674

**Published:** 2021-06-15

**Authors:** Albulena Sejdiu, Kristal N Pereira, Hajara Joundi, Yash R Patel, Sayeda A Basith, Victoria Ayala, Keerthika Mathialagan, Pradipta Majumder

**Affiliations:** 1 Psychiatry, Saints Cyril and Methodius University, Skopje, MKD; 2 Internal Medicine, Terna Medical College, Mumbai, IND; 3 Internal Medicine, University Cadi Ayyad, Faculty of Medicine and Pharmacy, Marrakesh, MAR; 4 Medicine, Kanak Hospital, Limdi, IND; 5 Psychiatry and Behavioral Sciences, Medical University of the Americas, Charlestown, KNA; 6 Psychiatry, Ross University School of Medicine, Bridgetown, BRB; 7 Psychiatry, Sree Balaji Medical College and Hospital, Chennai, IND; 8 Psychiatry, Drexel University College of Medicine, Philadelphia, USA; 9 Psychiatry, WellSpan Health, York, USA

**Keywords:** risk factors, prescription opioid, overdose prevention, mortality, substance recreational use, opioid use disorders

## Abstract

Objectives

To explore the demographic patterns of hospitalizations related to prescription opioid overdose (POD) and evaluate the mortality risk of association in POD inpatients.

Methodology

We conducted a cross-sectional study using the Nationwide Inpatient Sample of 184,711 POD inpatients. A binomial logistic regression model was used to evaluate the odds ratio (OR) of association for mortality risk due to comorbidities (substance use disorders (SUD) and medical complications) in POD inpatients.

Results

POD inpatients were majorly females (54.1%), older adults aged 51-75 years (48.5%), whites (81.5%), and from lower household income quartet (32.8%). The most prevalent comorbid SUD among POD inpatients was alcohol (15.7%), followed by cannabis (5.7%), cocaine (4.2%), and amphetamine (1.8%). Comorbid alcohol use disorders had a minimally increased association with mortality but were not statistically significant (OR = 1.036; P = 0.438). POD in patients with cardiac arrest had the highest risk of mortality (OR = 103.423; P < 0.001), followed by shock (OR = 15.367; P < 0.001), coma (OR = 13.427; P < 0.001), and respiratory failure (OR = 12.051; P < 0.001).

Conclusions

Our study indicates that the hospitalizations related to POD were more prevalent among females, elders between 51 and 75 years of age, whites, and those in the lower household income quartet. The prevalence of prescription opioid use and the hospitalization related to POD remains a significant public health issue. POD inpatients with medical complications were at a higher risk of mortality than with comorbid SUD.

## Introduction

Opioids are used to treat intractable severe pain disorders due to their potent analgesic effects [1]. However, we need to be cautious as opioids have abuse liability due to their tendency to activate the reward system [2]. The prescription of opioids carries the risk of misuse, abuse, diversion, addiction, and overdose-related deaths. Prescription opioid misuse or abuse behaviors may include taking opioids in higher doses or using them for other purposes than intended. Meanwhile, opioid overdose results in reduced respiratory drive [3]. Based on the agency for healthcare research and quality, opioid-related hospitalizations have increased by 64% between 2005 and 2014 in the United States [4]. Although the incidence of opioid use disorders in adults receiving prescriptions of opioids has drastically decreased in the past few years, rates of both fatal and nonfatal opioid overdose remain unchanged [5].

As the mortality related to substance use has increased significantly worldwide, more than 70% of these deaths are associated with opioid use [6]. According to the Centers for Disease Control and Prevention (CDC), approximately 500,000 deaths from opioid overdoses have been recorded from 1999 to 2019. In 2020, over 81,000 people in the United States died from an overdose, with the majority of deaths driven by synthetic opioid overdose [7]. While prescription opioids were responsible for opioid-related deaths in the 1990s, heroin and synthetic opioids were implicated in overdose-related deaths in the early 2010s [8].

Opioid-related overdose and abuse are common among men, the middle-aged population, and those of low socioeconomic status [2]. A secondary analysis of the data of Mortality Disparities in American Communities study demonstrated that males, whites, American Indians, widowed, and unemployed were associated with a higher risk of death due to opioid overdose [9]. Chronic pain, depression, anxiety, sleep disorders, concomitant use of benzodiazepines, and other medical comorbidities are associated with a greater risk of opioid abuse and overdose [10].

Beyond an individual’s problematic use behaviors, prescription behaviors may be an added contribution to the risk of mortality. Based on a study involving 2.7 million US adolescents and young adults, daily opioid dosages and extended-release, long-acting opioid use were associated with higher mortality risk related to prescription overdose [11]. Additionally, a higher mortality rate was also found among those receiving both regularly scheduled and as-needed scheduled opioid prescriptions [12].

However, even though the rates of opioid prescription have significantly reduced, mortality due to highly potent and illicit opioid use continues to increase [13]. Thus, we conducted a cross-national study to explore the demographic pattern for prescription opioid overdose (POD) hospitalizations and to evaluate the mortality risk of association due to substance use disorders (SUD) and medical complications in POD inpatients.

## Materials and methods

Study sample

Our study included 184,711 inpatients (age 6 to 75 years) with a primary discharge diagnosis of POD using the Nationwide Inpatient Sample (NIS). The NIS is the largest inpatient database and represents a sample of 4,411 non-federal community hospitals across 44 states in the United States [14].

Variables

The demographic variables included in this study were age at admission, sex, race, and median household income. We defined comorbidities as any co-existing SUD (alcohol, cannabis, cocaine, and amphetamine) and other medical complications (encephalitis, meningitis, infective endocarditis, coma, respiratory failure, cardiac arrest, and shock). The hospitalization outcomes of interest included in-hospital mortality, reported as all-cause in the NIS [15].

Statistical analysis

We delineated the pattern of demographic characteristics and comorbidities in POD inpatients by performing descriptive statistics. Next, we measured the trend and overall in-hospital mortality seen in POD inpatients using cross-tabulation and linear-by-linear association tests. A binomial logistic regression model was used to evaluate the odds ratio (OR) of association for mortality risk among comorbidities (SUD and medical complications) in POD inpatients. All analyses were conducted using the Statistical Package for the Social Sciences (SPSS) version 26.0 (IBM Corp., Armonk, NY), and statistical significance was set at a two-sided P-value of <0.01.

Ethical approval

The NIS is a publicly available de-identified dataset with the protection of patients, physicians, and hospital-related information; hence, we were not required to take institutional review board approval for our study [14].

## Results

Our study population included 184,711 POD inpatients who were predominantly females (54.1%). We observed higher rates of POD among the 51-75-year olds (48.5%), followed by adults aged 36-50 years (27.2%) and adults aged 18-35 years (21.9%). POD-related hospitalizations were prevalent among whites (81.5%), followed by blacks (8.7%) and Hispanics (6.4%). The rates also differed by socioeconomic status as patients in the lower household income quartet had higher hospitalization rates (32.8%), while the higher household income quartet had the lowest rates (16.2%), as shown in Table 1.

**Table 1:** Demographic characteristics of POD-related hospitalizations. POD: prescription opioid overdose

Variable	%
Age at admission, in years	
6–17	2.4
18–35	21.9
36–50	27.2
51–75	48.5
Sex	
Male	45.9
Female	54.1
Race	
White	81.5
Black	8.7
Hispanic	6.4
Others	3.5
Median household income, in percentile	
0–25^th^	32.8
26^th^–50^th^	27.6
51^st^–75^th^	23.5
76^th^–100^th^	16.2

Among POD inpatients, the most prevalent comorbid SUD was alcohol (15.7%), followed by cannabis (5.7%), cocaine (4.2%), and amphetamine (1.8%). Comorbid alcohol use disorder had a minimally increased association with mortality but was not statistically significant (OR = 1.036; P = 0.438). Other comorbid SUD had a statistically significant negative association with mortality.

POD inpatients with medical complications had a higher risk of mortality. The most prevalent medical complications were respiratory failure (33.7%), coma (14.8%), and shock (3.4%). Though the prevalence of cardiac arrest was 2%, it was associated with the highest risk of mortality (OR = 103.423; P < 0.001), followed by shock (OR = 15.367; P < 0.001), coma (OR = 13.427; P < 0.001), and respiratory failure (OR = 12.051; P < 0.001). Although the prevalence of meningitis was very low (0.1%), it was significantly associated with a higher mortality risk (OR = 4.216; P < 0.001), as shown in Table 2.

**Table 2:** Mortality risk in POD-related hospitalizations by comorbidities. POD: prescription opioid overdose

Comorbidity	%	Logistic regression analysis and mortality		
		Odds ratio	95% Confidence interval	P-value
Substance use disorders				
Alcohol	15.7	1.036	0.947–1.133	0.438
Cannabis	5.7	0.702	0.598–0.824	<0.001
Cocaine	4.2	0.715	0.592–0.863	<0.001
Amphetamine	1.8	0.433	0.301–0.621	<0.001
Medical complications				
Encephalitis	0.1	1.177	0.467–2.963	0.730
Meningitis	0.1	4.216	2.201–8.079	<0.001
Infective endocarditis	0.1	1.985	0.772–5.102	0.155
Coma	14.8	13.427	12.501–14.421	<0.001
Respiratory failure	33.7	12.051	10.979–13.228	<0.001
Cardiac arrest	2.0	103.423	95.024–112.563	<0.001
Shock	3.4	15.367	14.228–16.597	<0.001

## Discussion

Our study found that POD hospitalizations were prevalent in females (54.1%), elders in the 51-75-year age group (48.5%), whites (81.5%), and those in the lower household income quartet (32.8%). Past studies reflect our results where whites (67%) and those from low-income families had a higher likelihood for POD-related hospitalization [16]. Opioid prescription is more prevalent among whites because there is an unconscious bias resulting in under-prescription towards racial/ethnic minorities. There exists an inclination towards lower-income quartet regarding POD-related hospitalizations. Even though prescription opioids are much more expensive than other illicit substances, they may be less of a barrier for individuals who qualify for Medicaid or programs like supplemental security income with Medicaid benefits. Thus, low-income individuals have easier access to pain clinics and prescription opioids [17]. The rate of hospitalization for females has transcended that for males regarding POD [4]. This preponderance could be attributed to the difference in pain sensitivity between both sexes [18]. Although there are neurobiological differences between the two, there also exists a psychosocial disadvantage among women. Societal gender roles, differences in coping mechanisms, and a higher frequency of comorbid anxiety and depression could lead to the differences in women. Men are also less likely to seek help from healthcare professionals, which may contribute to women being more likely to be prescribed and to use prescription opioids [19].

Since 2005, opioid-related hospitalizations have increased dramatically [4]. Past studies have shown a higher odds of association with POD-related hospitalizations in pediatric inpatients with comorbid SUD [16]. A past study found that overdose among children correlated to accidental or unintentional overdose from parental opioid prescriptions [20]. Prescription opioids can also serve as an impetus to heroin abuse, thereby increasing the susceptibility to other SUD [21]. A socioeconomic impediment is associated with increased hospitalization due to POD. This finding of our study calls attention to the need for greater focus on incongruity in addiction care and treatment outcomes. The mortality associated with opioid use disorder is 10-fold higher than in the general population [22]. National drug overdose deaths involving prescription opioids increased from 3,442 in 1999 to 17,029 in 2017, and from 2017 to 2019, the deaths reduced to 14,139 [23].

Among POD inpatients, the most prevalent comorbid SUD was alcohol (15.7%) which had a nonsignificant association with in-hospital mortality when controlled for other medical complications and potential confounders. Our finding is similar to a previous survey that found prescription opioid use to be commonly associated with alcohol and cannabis use disorders [24]. Increased mortality within this subgroup in previous studies could be due to the higher prevalence of other comorbid SUD and high-risk behaviors. Opioid users who drink heavily are likely to develop a more severe liver injury, progress to cirrhosis, and have an increased risk for liver cancer and mortality. High-risk behaviors could also place them at risk of infections such as hepatitis C. Hence, comorbid SUDs and comorbidities could have acted as confounders for the mortality association between POD and alcohol use [25].

In our study, POD inpatients with medical complications had a higher risk of mortality. Moreover, about half of our study inpatients were older adults (age 51-75 years). Opioids bind to mu and delta receptors in the central nervous system, depressing the respiratory center and increasing the cerebral partial pressure of carbon dioxide. In POD, patients may not be able to compensate for respiratory depression and go into respiratory failure which is the possible reason why about one-third of our POD inpatients had respiratory failure. Additionally, opioid use is associated with elevated blood pressure, body mass index, glycated hemoglobin, lower calcium, and lower high-density lipoprotein, all of which have poor cardiovascular outcomes [26]. These findings are consistent with our finding of cardiac arrest having the highest mortality risk in POD patients by 103 times. Long-acting opioids also cause serious infections such as pneumonia, meningitis, encephalitis, and septicemia [27]. In our study, although the prevalence of meningitis was very low (0.1%), it was significantly associated with four times higher in-hospital mortality risk.

As mentioned earlier, prescribing behaviors may also contribute to the risk of mortality associated with POD. An analysis from the CDC showed that although prescription rates have dropped by 20% between 2015 and 2017, current levels remain almost three times higher than they were in 1999 [28]. The National Institutes of Health is working towards achieving safe, effective, and nonaddictive strategies to manage uncontrolled chronic pain. The Center for Substance Abuse and Mental Health Services has implemented “the prescription behavior surveillance system” to identify and control the opioid overdose epidemic by monitoring and comparing trends with opioid prescriptions between states. These interventions could provide better control and prevention of opioid abuse or misuse [29]. Besides enhanced public health surveillance, the US Department of Health and Human Services has been focusing on increasing access to treatment and recovery services, encouraging the use of overdose-reversing drugs, and establishing specific protocols for pain management [30].

This study has few limitations due to the administrative nature of the NIS. It lacks patient-level clinical information, and inpatients are grouped based on the diagnostic codes, which may lead to the under-reporting of comorbidities. Besides, the data were limited to non-federal community-based hospitals and excluded inpatients at the state and county psychiatric hospitals. One of the strengths of our study is the NIS population-based inpatient representation of associations between diseases and comorbidities. The chances of recall bias are minimal. Our study reflects a large nationally representative sample which also increases the power of our findings. The study has also explored an important but less commonly explored area of research. Further studies should focus on the preventative aspect of deaths related to POD.

## Conclusions

Our study indicates POD-related hospitalizations were more prevalent among females, elders between 51 and 75 years of age, whites, and those in the lower household income quartet. The prevalence of prescription opioid use and POD-related hospitalization remains a significant public health issue. POD inpatients with medical complications including cardiac arrest (increased by 103 times), shock (increased by 15 times), coma (increased by 13 times), and respiratory failure (increased by 12 times) were at a higher risk of in-hospital mortality than with comorbid SUD.
